# Supplementary material for: Evidence supported by Mendelian randomization: impact on inflammatory factors in knee osteoarthritis
Source: Front Med (Lausanne). 2024 May 28;11:1382836. doi: 10.3389/fmed.2024.1382836 (PMC11165061; doi:10.3389/fmed.2024.1382836)
Supplement: Supplementary file 1 [file Data_Sheet_1.ZIP › data/GCST90274795_5e6/795.leaveoneout.pdf]

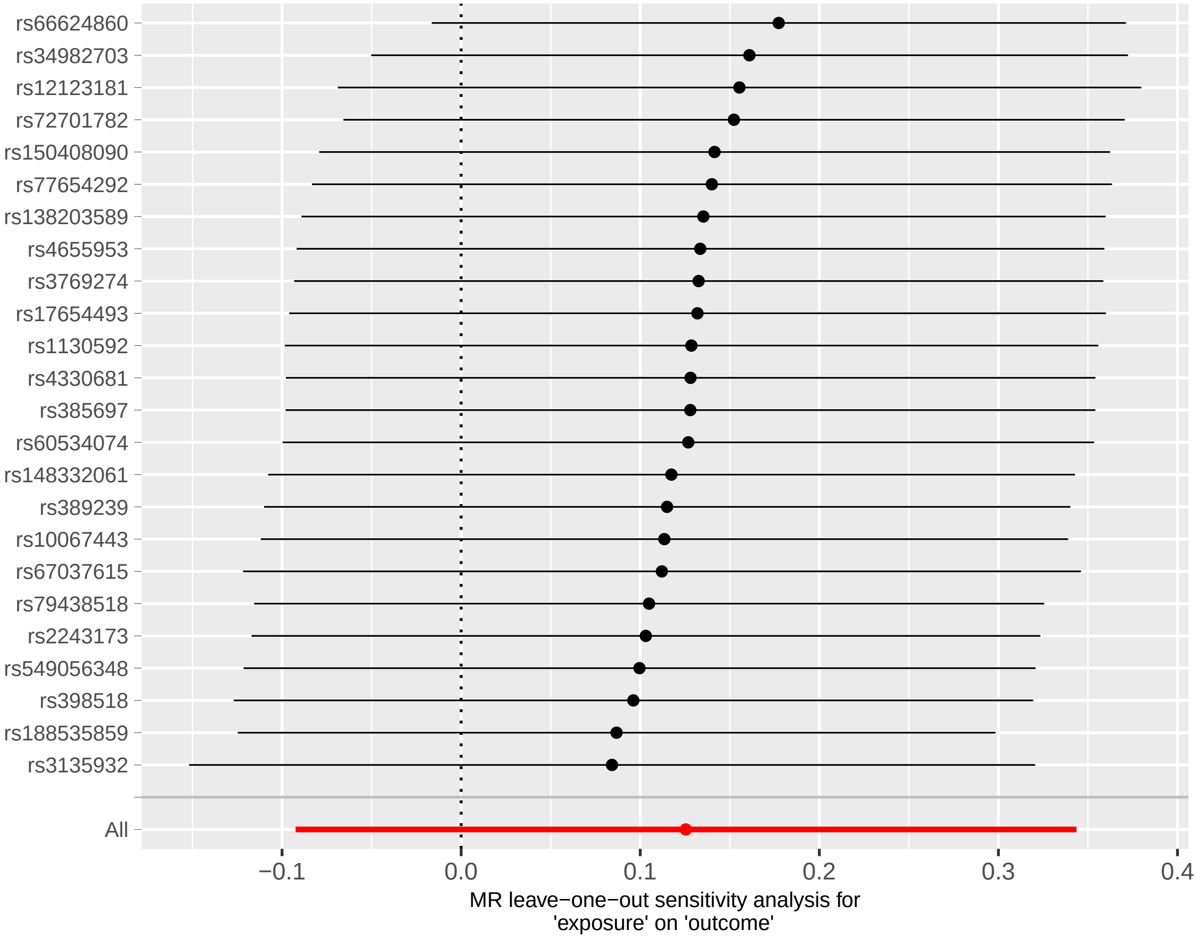

rs66624860
rs34982703
rs12123181
rs72701782
rs150408090
rs77654292
rs138203589
rs4655953
rs3769274
rs17654493
rs1130592
rs4330681
rs385697
rs60534074
rs148332061
rs389239
rs10067443
rs67037615
rs79438518
rs2243173
rs549056348
rs398518
rs188535859
rs3135932
All
−0.1
0.0
0.1
0.2
0.3
0.4
MR leave−one−out sensitivity analysis for
'exposure' on 'outcome'
